# Morphological and molecular detection of *Hepatozoon* species in amphibians and reptiles from Mato Grosso, Midwest Brazil

**DOI:** 10.1590/S1984-29612025076

**Published:** 2026-02-02

**Authors:** Victória Luiza de Barros Silva, Ana Clécia dos Santos Silva, José Nilton de Araujo Gonçalves, Natália Paludo Smaniotto, Letícia Pereira Úngari, Rachel de Sousa Lima Pulcherio, Juliano Bortolini, Rosa Helena dos Santos Ferraz, Christine Strüssmann, Thállitha Samih Wischral Jayme Vieira, Rafael Felipe da Costa Vieira, Richard de Campos Pacheco

**Affiliations:** 1 Universidade Federal de Mato Grosso – UFMT, Faculdade de Medicina Veterinária – FAVET, Programa de Pós-graduação em Ciências Veterinárias – PPGVET, Cuiabá, MT, Brasil; 2 University of North Carolina at Charlotte, Department of Bioinformatics, Charlotte, NC, USA; 3 University of North Carolina, Center for Computational Intelligence to Predict Health and Environmental Risks, Charlotte, USA; 4 Universidade Federal de Mato Grosso – UFMT, Programa de Pós-graduação em Zoologia, Cuiabá, MT, Brasil; 5 Universidade Federal de Mato Grosso – UFMT, Instituto de Ciências Naturais, Humanas e Sociais, Núcleo de Estudos da Biodiversidade da Amazônia Matogrossense/NEBAM, Sinop, MT, Brasil; 6 Universidade de São Paulo – USP, Instituto de Ciências Biomédicas, Departamento de Parasitologia, Laboratório de Taxonomia e Filogenia de Tripanossomatídeos, São Paulo, SP, Brasil; 7 University of North Carolina at Charlotte, Department of Chemistry, Charlotte, NC, USA; 8 University of North Carolina at Charlotte, Department of Epidemiology and Community Health, Charlotte, NC, USA.

**Keywords:** Apicomplexa, Brazilian Herpetofauna, Hemogregarines, Pantanal, Brazilian Savanna, Apicomplexa, Herpetofauna Brasileira, Hemogregarinas, Pantanal, Cerrado Brasileiro

## Abstract

The *Hepatozoon* genus is composed of hemoparasites widely distributed, though their diversity and phylogeny remain poorly understood in the Brazilian herpetofauna. We aimed to characterize *Hepatozoon* species infecting the blood and spleen of amphibians and reptiles in Mato Grosso, Midwestern Brazil, using morphological and molecular tools. In total, 146 animals from 19 species (8 amphibians and 11 reptiles) were evaluated. Giemsa-stained blood smears revealed *Hepatozoon*-like inclusions in 35 individuals (8 amphibians and 27 reptiles), including new infection records in the anurans *Boana raniceps* and *Trachycephalus typhonius*, and the snake *Eunectes notaeus*. Morphological analysis revealed diverse morphotypes. Molecular analysis of the 18S rRNA gene identified six haplotypes in *Rhinella diptycha*, *Ameiva ameiva*, *Boa constrictor*, *Epicrates crassus*, *E. notaeus*, and *Caiman yacare*. Phylogenetic analyses revealed clustering with clades linked to *H. musa* and *H. caimani*, suggesting the presence of potentially novel lineages. These findings highlight the high genetic diversity of *Hepatozoon* in the region and emphasize the value of integrative approaches in parasitological research.

## Resumo

O gênero *Hepatozoon* é composto por hemoparasitos amplamente distribuídos, embora sua diversidade e filogenia permaneçam pouco compreendidas na herpetofauna brasileira. Nosso objetivo foi caracterizar as espécies de *Hepatozoon* que infectam o sangue e o baço de anfíbios e répteis no Mato Grosso, Centro-Oeste do Brasil, utilizando ferramentas morfológicas e moleculares. No total, 146 animais de 19 espécies (8 anfíbios e 11 répteis) foram avaliados. Esfregaços de sangue corados com Giemsa revelaram inclusões semelhantes a *Hepatozoon* em 35 indivíduos (8 anfíbios e 27 répteis), incluindo novos registros de infecção nos anuros *Boana raniceps* e *Trachycephalus typhonius*, e na serpente *Eunectes notaeus*. A análise morfológica revelou diversos morfotipos. A análise molecular do gene 18S rRNA identificou seis haplótipos em *Rhinella diptycha*, *Ameiva ameiva*, *Boa constrictor*, *Epicrates crassus*, *E. notaeus* e *Caiman yacare*. As análises filogenéticas revelaram agrupamentos com clados ligados a *H. musa* e *H. caimani*, sugerindo possíveis novas linhagens. Esses achados destacam a alta diversidade genética de *Hepatozoon* na região e enfatizam o valor de abordagens integrativas na pesquisa parasitológica.

## Introduction

The state of Mato Grosso is one of the largest in Brazil, featuring exceptional biodiversity and a distinct climate, which together contribute to the presence of several unique ecosystems (Nunes et al., 2017). These ecological characteristics, compounded by environmental changes, may increase the emergence of diseases in new hosts, as such changes alter ecosystem structure and influence the transmission and spread of pathogens (Reis et al., 2021). Moreover, ecological interactions are continually shifting due to multiple factors, making parasitism a dynamic and successful ecological strategy capable of modulating host communities (Timi & Poulin, 2020).

The phylum Apicomplexa is the only major taxonomic group composed entirely of parasitic organisms and is therefore of significant interest to parasitologists. However, despite its relevance, Apicomplexa remains one of the least understood in terms of biodiversity (Morrison, 2009). Within this phylum, the genus *Hepatozoon* (Apicomplexa: Hepatozoidae) includes protozoan parasites widely distributed among terrestrial vertebrates and their hematophagous invertebrate vectors (Smith, 1996). It is one of six hemogregarine genera, all of which exhibit heteroxenous life cycles involving a vertebrate intermediate host and an invertebrate definitive host. Infections with *Hepatozoon* are frequently observed in reptiles and amphibians, although they remain underdiagnosed, particularly in South America (Moço et al., 2002; Úngari et al., 2018). Transmission can occur through the ingestion of infected arthropods, vertical transmission, and trophic interactions involving predator-prey dynamics (Tomé et al., 2012; Kauffman et al., 2017; Schäfer et al., 2022). Despite this ecological complexity, the taxonomy and phylogeny of *Hepatozoon* remain poorly resolved, especially in the Neotropics.

Research on *Hepatozoon* infections in the Brazilian herpetofauna still faces significant gaps concerning host range, vector species, and geographic distribution (Úngari et al., 2018; Ferreira et al., 2020; Picelli et al., 2020). Several studies have described *Hepatozoon* spp. infecting reptiles—including snakes (Moço et al., 2002; O'Dwyer et al., 2013; Borges-Nojosa et al., 2017; Paula et al., 2022; Úngari et al., 2022), crocodilians (Viana et al., 2010; Clemente et al., 2023; Úngari et al., 2024), and lizards (Harris et al., 2015; Lima et al., 2021; Morais et al., 2024)—as well as anurans (Leal et al., 2015; Ferreira et al., 2020; Úngari et al., 2021). Nonetheless, the number of species with complete data—including morphometric, molecular, and phylogenetic characterization—remains low (Picelli et al., 2020; Úngari et al., 2022). This lack of comprehensive data makes it difficult to accurately describe species and leads to identifications based solely on microscopic or molecular evidence, often resulting in conflicting classifications.

Relying exclusively on morphology for parasite identification presents challenges, such as inconsistent specimen preservation, trait plasticity due to environmental or host factors, and morphological convergence. These factors can obscure true relationships among species and complicate classification (Smith, 1996). On the other hand, overreliance on molecular tools can lead to premature species designations or synonymies, as it tends to disregard valuable morphological traits. This imbalance has caused taxonomic instability and misidentification in several parasite groups (Koukol & Delgado, 2021). Additionally, the use of single-locus barcoding, especially the 18S ribosomal RNA (18S rRNA) gene, has often yielded conflicting results between morphology-based and molecular classifications, limiting species delimitation (Morrison, 2009).

Molecular methods have become indispensable in recent decades, with “molecular morphology” emerging as a promising tool to address gaps left by traditional morphology (Tessler et al., 2022). However, studies on hepatozoids still depend almost exclusively on the 18S rRNA gene—a highly conserved marker with limited resolution for distinguishing cryptic or closely related lineages (Nadler & de León, 2011; Morrison, 2009). As a result, this marker constrains both taxonomic comparisons and the construction of robust phylogenies. Because 18S rRNA evolves slowly, it often fails to differentiate species that exhibit subtle morphological or ecological differences. While it is useful for genus-level identification, its limited discriminatory power becomes problematic in regions with high intraspecific or intraregional diversity. Recent phylogenomic research has emphasized the value of multilocus approaches in clarifying evolutionary relationships and uncovering cryptic diversity (Reck et al., 2025).

In this context, considering the ecological complexity of Mato Grosso and the scarcity of integrated data, this study employed an integrative approach combining morphological, morphometric, molecular, and phylogenetic analyses to investigate the diversity of *Hepatozoon* infecting amphibians and reptiles in this Brazilian state.

## Materials and Methods

### Study area and sampling

**Figure 1:**
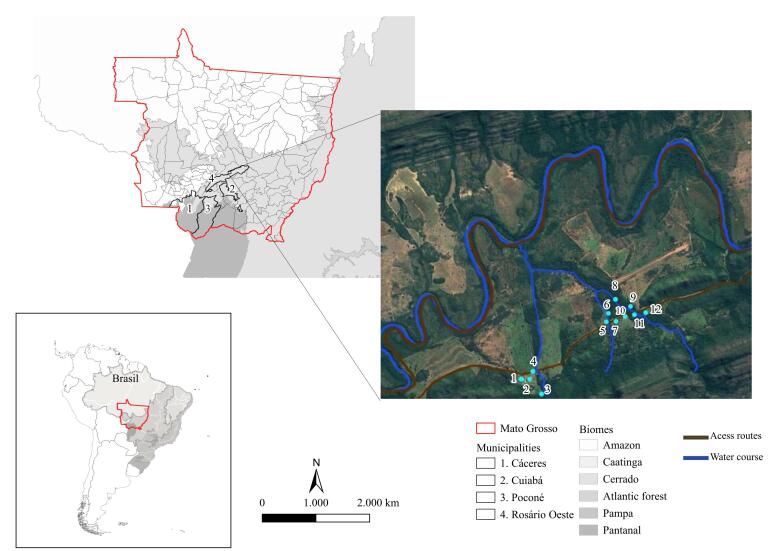
Map of municipalities within the state of Mato Grosso, Brazil, indicating the origins of reptiles referred to the Veterinary Hospital of the Federal University of Mato Grosso between 2021 and 2023. The numbered localities (1–12), marked with light blue dots, represent amphibian and reptile sampling sites in Rosário Oeste municipality during 2022.

This study employed two sampling strategies. First, free-living and captive reptiles from the municipalities of Cáceres, Cuiabá, and Poconé (Figure 1) admitted to the Veterinary Hospital at the Federal University of Mato Grosso (UFMT), Brazil, between 2021 and 2023, were evaluated. The animals were admitted to the hospital due to a variety of clinical conditions, including trauma, burns, fractures, and other injuries. Additionally, amphibians and reptiles were captured during field expeditions conducted in the SESC Serra Azul State Park, municipality of Rosário Oeste (Figure 1).

Animals were captured using pitfall traps (Strüssmann, 2000; Rocha & Prudente, 2010). Sampling points were distributed parallel to the stream in gallery forest environments and perpendicular in dry semi-deciduous forest environments. To ensure sampling independence, all points were spaced at least 100 m apart (Campbell & Christman, 1982). At each sampling site, two 60-L plastic buckets were buried on either side of a 15-m-long plastic drift fence (0.5 m height), spaced 10 m apart and supported by wooden stakes. To reduce thermal stress, bucket lids were suspended above each trap using metal stakes. To prevent water accumulation and drowning during rainfall, 3 mm holes were drilled in the bucket bottoms, and Styrofoam plates were placed inside. During the dry season, moistened fabric scraps were added to maintain humidity. Sampling points were checked daily. Twelve field expeditions, each lasting seven consecutive days, were conducted at 12 collection points in the park in 2022.

Specimen identification followed standard guides and taxonomic keys for amphibians (Narvaes & Rodrigues, 2009; Lavilla & Brusquetti, 2018; Ávila et al., 2021; Pereyra et al., 2021) and reptiles (Ferrarezzi & Monteiro, 2001; Marques et al., 2015; Entiauspe-Neto et al., 2020; Guillory et al., 2020). Captured or hospitalized individuals were weighed using either a Pesola^®^ hand-held dynamometer (Switzerland) or a digital scale. Maximum blood volume collected was limited to 1% of body mass (Campbell & Grant, 2022).

Blood samples were obtained via venipuncture (amphibians: abdominal or femoral vein; reptiles: caudal or abdominal vein; snakes: paravertebral or caudal vein). Smears were prepared immediately, air-dried, fixed in absolute methanol, and stained with Giemsa (Rosenfeld, 1947). Residual blood was stored in cryotubes and frozen at –20 °C for later DNA extraction.

Common, non-threatened amphibian species from the families Bufonidae (*e.g.*, *Rhinella diptycha*, *R. major*), Hylidae (*e.g.*, *Boana raniceps*), and Leptodactylidae (*e.g.*, *Leptodactylus labyrinthicus*, *Physalaemus nattereri*) were euthanized using chemical methods (Close et al., 1997; Wright, 2010). Blood was collected by cardiac puncture following sedation with 1–2% lidocaine (2 mg/kg, intramuscular). Reptiles were euthanized via intramuscular injection of sodium thiopental (60–100 mg/kg) (Close et al., 1997; Diethelm, 2010), and spleen fragments were obtained via laparotomy, stored in cryotubes, and frozen at –20 °C for molecular analyses. Specimens were deposited at the Coleção Zoológica da Universidade Federal de Mato Grosso (UFMT), Cuiabá, Brazil.

### Morphological and morphometric analysis

Blood smears were examined using a Leica DM 500 microscope (Leica Microsystems, Heerbrugg, Switzerland) at total magnifications of ×400 and ×1000. Images were captured using a digital Leica camera and processed in LAS V4.8 software (Leica Microsystems). Infection rate was estimated by counting *Hepatozoon* inclusions in 2,000 erythrocytes across 20 replicates of 100 cells (Picelli et al., 2020).

Parasite length, width, and area were measured using ImageJ (v1.47), and means, ranges, and standard deviations were calculated in micrometers (μm). Parasite developmental stages (immature gamont, mature gamont, macrogamont, microgamont) were identified based on established criteria (Telford, 2010; Al-Quraishy et al., 2021). Morphotypes were categorized by morphological features (curvature, nucleus position, host cell distortion) and quantitative metrics.

Photomicrographs were taken for all morphotypes and developmental stages. Measurements for each morphotype were summarized using descriptive statistics and presented in μm.

### Statistical analysis

Only mature gamonts—those observed in at least 10 erythrocytes—were selected for analysis to minimize variability related to life stage or detection limits. Morphological features (area, perimeter, maximum/minimum Feret diameters) were analyzed using descriptive and multivariate statistics. For each morphotype, summary metrics included sample size, mean, standard deviation, coefficient of variation, and interquartile range. To assess sample homogeneity and detect outliers, Mahalanobis distance was calculated at a 5% significance level (Fisher, 1925). This metric also evaluated whether individuals from the same host species shared a common morphotype. Analyses were conducted in R software (R Core Team, 2024).

### Molecular and phylogenetic analysis

DNA was extracted from 100 μL of whole blood or 5 mg of spleen using the phenol-chloroform method with isopropanol precipitation (Sambrook & Russell, 2001). Extraction was monitored by conventional PCR targeting the endogenous vertebrate small subunit ribosomal RNA (16S rRNA) gene (Palumbi et al., 1991). Ultrapure water served as a negative control for contamination.

Subsequently, all DNA samples were screened using a previously described PCR assay targeting a fragment (600 base pairs -bp) of the *Hepatozoon* 18S rRNA gene (Ujvari et al., 2004). For species characterization, *Hepatozoon*-positive samples were subjected to an additional PCR protocol targeting a 1,346 bp fragment of the 18S rRNA gene. This amplification protocol utilizes a forward primer to *Hepatozoon* species (Perkins & Keller, 2001) and a reverse primer to *Haemogregarina* species (Ujvari et al., 2004). These primers target overlapping and conserved regions of the 18S rRNA gene among apicomplexan hemoparasites (Abdel-Baki et al., 2020).

Reactions were performed in 25 μL with 0.4 mM primers, 1× GoTaq^®^ G2 Master Mix (Promega), 5 μL DNA, and nuclease-free water. A synthetic double-strand DNA fragment (gBlock™, IDT, Coralville, IA, USA) with a 10bp deletion was designed as the positive control, using the *Hepatozoon canis* sequence (GenBank^®^ accession no. MH615006) as a reference. Nuclease-free water was used as the negative control. PCR products were run in 1.5% agarose gels at 100 V, visualized under UV light, cloned using pGEM^®^-T Easy Vector (Promega), and purified using QIAprep Spin Miniprep Kit (Qiagen). Sanger sequencing was conducted, and sequences were assembled in Geneious Prime v2025.0.3 (Biomatters, USA).

Phylogenetic reconstruction was performed via Bayesian inference using BEAST 1.8.0 (Drummond et al., 2012). Three independent MCMC runs of 100 million generations were sampled every 10,000 generations with a 10% burn-in. The GTR + G model was selected using AIC in jModelTest 2.1.10 (Darriba et al., 2012). We assessed Effective Sample Size (ESS) of the tree using Tracer v1.7.2. All estimated parameters had Effective Sample Size (ESS) values greater than 200, indicating excellent sampling and mixing of the MCMC. Trees were visualized in FigTree v1.4.4 (Rambaut et al., 2016) and finalized in Inkscape v0.92.2. *Neospora caninum* (GenBank^®^: U03069) was used to root the tree.

## Results

### Animal sampling, blood collection, and tissue collection

A total of 146 animals were evaluated: 34 reptiles admitted to the Veterinary Hospital at the Federal University of Mato Grosso (UFMT) and 112 specimens (69 anurans and 43 reptiles) captured during field expeditions. Capture efforts totaled 96,768 bucket-hours (24 h per bucket × 48 buckets × 84 sampling days) in the municipality of Rosário Oeste.

In total, eight anuran species and 77 reptiles representing 11 species were included in the study. Data on species, number of individuals, municipality, and origin are summarized in Table 1. Moreover, 146 blood samples and 26 spleen samples were collected from euthanized individuals: *B. raniceps* (n = 8), *L. macrosternum* (n = 10), *T. typhonius* (n = 2), *L. labyrinthicus* (n = 2), *O. taurinus* (n = 2), and *R. diptycha* (n = 2).

**Table 1:** Relationships among specimens, blood smears, and PCR assay results from free-living (FL) and captive (C) amphibians and reptiles in the state of Mato Grosso, Brazil, between 2021 and 2023.

	Species	No. of individuals	Municipality	Origin	Mean parasitemia (%)	Results of individuals who tested PCR-positive for *Hepatozoon* sp.^1^/ Total no. of individuals tested	
						**Blood**	**Spleen**
**Anura**							
**Hylidae**							
	*Boana raniceps*	11	Rosário Oeste	FL	4	0/9 (0)	0/6 (0)
	*Osteocephalus taurinus*	3	Rosário Oeste	FL	-	1/3 (33.3%)	1/2 (50%)
	*Pseudis platensis*	7	Rosário Oeste	FL	-	0/6 (0)	
	*Trachycephalus typhonius*	7	Rosário Oeste	FL	2	2/7 (28.5%)	1/2 (50%)
**Bufonidae**							
	*Rhinella diptycha*	12	Rosário Oeste	FL	2	1/11 (9%)	0/1 (0)
**Leptodactylidae**							
	*Leptodactylus labyrinthicus*	4	Rosário Oeste	FL	4	1/4 (25%)	0/2 (0)
	*Leptodactylus macrosternum*	18	Rosário Oeste	FL	4	5/17 (29.4%)	4/10 (40%)
	*Leptodactylus syphax*	7	Rosário Oeste	FL	-	2/7 (28.5%)	-
**Squamata**							
**Hoplocercidae**							
	*Hoplocercus spinosus*	1	Rosário Oeste	FL	-	0/1 (0)	-
**Scincidae**							
	*Copeoglossum nigropunctatum*	1	Rosário Oeste	FL	-	0/1 (0)	-
**Teiidae**							
	*Ameiva ameiva*	11	Rosário Oeste	FL	2	1/10 (10%)	-
	*Iguana iguana*	4	Cuiabá	FL	-	0/4 (0)	-
**Tropiduridae**							
	*Tropidurus lagunablanca*	2	Rosário Oeste	FL	-	0/2 (0)	-
	*Tropidurus torquatus*	3	Cuiabá	FL	-	0/3 (0)	-
**Boidae**							
	*Boa constrictor*	1	Rosário Oeste	FL	45	1/1 (100%)	-
		7	Cáceres	FL	35	0/2 (0)	-
		7	Cuiabá	C	20	3/7 (42.8%)	-
		10	Cuiabá	FL	15	-	-
		3	Poconé	FL	40	1/2 (50%)	-
	*Eunectes notaeus*	2	Cáceres	FL	60	1/2 (50%)	-
		6	Cuiabá	FL	30	1/5 (20%)	-
		7	Poconé	FL	80	1/4 (25%)	-
	*Epicrates crassus*	2	Rosário Oeste	FL	30	2/2 (100%)	-
**Dipsadidae**							
	*Erytrolampus poecilogyrus*	1	Cuiabá	FL	-	0/1 (0)	-
**Crocodylia**							
**Alligatoridae**							
	*Caiman yacare*	6	Cáceres	FL	60	4/5 (80%)	-
		3	Cuiabá	C	30	0/3 (0)	-
**Total**		**146**			**-**	**27/119**	**6/23**

1 Based on the PCR protocol described by Ujvari et al. (2004).

### Morphological and morphometric analysis

**Figure 2:**
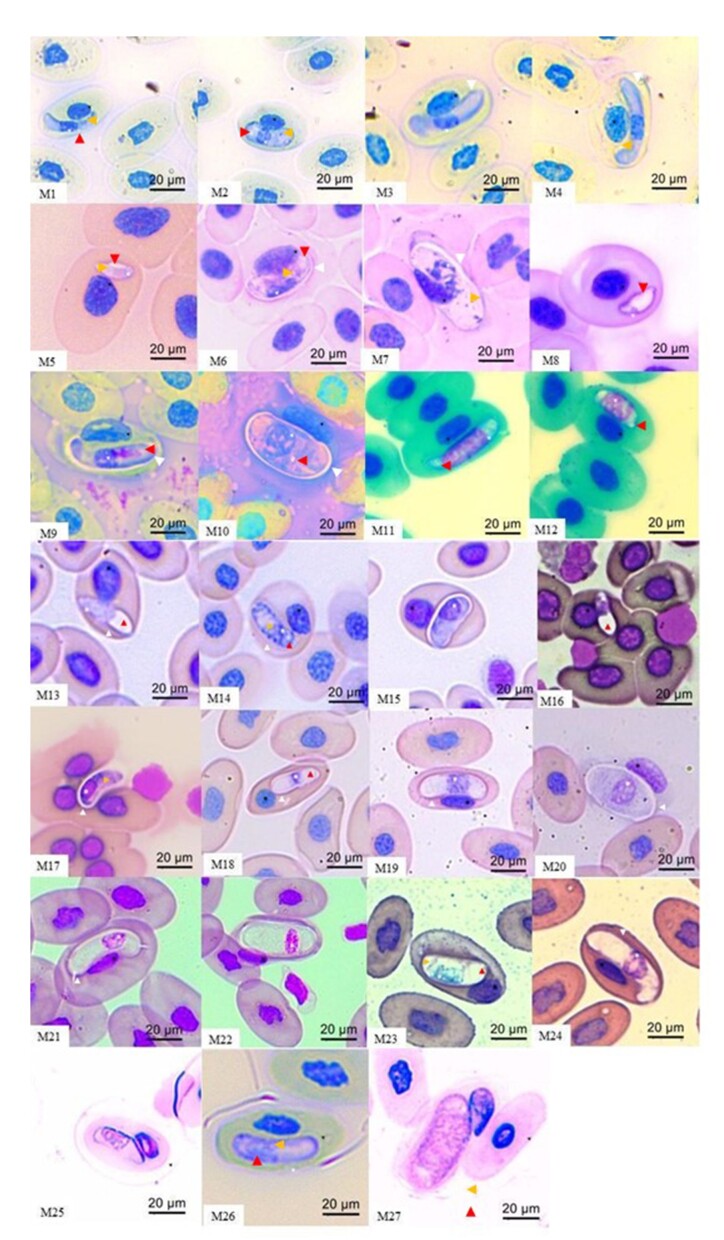
Morphotypes of *Hepatozoon* spp. identified in amphibians and reptiles from Mato Grosso. In *Boana raniceps*: M1 and M2 are immature gamonts; M3 and M4 are mature gamonts. In *Trachycephalus typhonius*: M5 (immature gamont), M6 (mature gamont), and M7 (macrogametocyte). In *Rhinella diptycha*: M8 (immature gamont), M9 (mature gamont), and M10 (macrogametocyte). In *Leptodactylus labyrinthicus*: M11 and M12 (immature gamonts). In *Leptodactylus macrosternum*: M13 (immature gamont), M14 (mature gamont), and M15 (macrogametocyte). In *Ameiva ameiva*: M16 and M17 (immature gamonts). In *Boa constrictor*: M18 (immature gamont), M19 (mature gamont), and M20 (macrogamont). In *Eunectes notaeus*: M21 (mature gamont) and M22 (macrogamont). In *Epicrates crassus*: M23 (immature gamont) and M24 (mature gamont). In *Caiman yacare*: M25 (immature gamont), M26 (mature gamont), and M27 (macrogamont). Structures are identified as follows: red blood cell nucleus (black asterisk), parasite nucleus (white asterisk), vacuole (red arrowhead), basophilic granules (yellow arrowhead), and capsule (white arrowhead).

A total of 35 out of 146 animals (23.9%) tested positive for *Hepatozoon*-like parasites by blood smear, comprising eight amphibians and 27 reptiles. Positive amphibians included: *B. raniceps* (n = 1), *T. typhonius* (n = 2), *R. diptycha* (n = 1), *L. labyrinthicus* (n = 1), and *L. macrosternum* (n = 3). Among reptiles, positive individuals included: *A. ameiva* (n = 2), *B. constrictor* (n = 9), *E. notaeus* (n = 10), *E. crassus* (n = 2), and *C. yacare* (n = 4). In total, 27 distinct morphotypes were described (Figure 2).

Parasitemia levels ranged from 2 gamonts per 100 cells (e.g., *B. raniceps*, *T. typhonius*, *R. diptycha*, and *A. ameiva*) to 80 per 100 cells in *E. notaeus*. All morphometric data are presented in Supplementary Table 1. Due to low parasitemia and limited visibility of forms, morphological identification was restricted to the genus *Hepatozoon* in *B. raniceps*, *T. typhonius*, *R. diptycha*, *L. labyrinthicus*, *L. macrosternum*, and *A. ameiva*. In contrast, morphotypes from *B. constrictor*, *E. crassus*, *E. notaeus* and *C. yacare* showed similarity to *Hepatozoon cevapii*, *H. musa*, *H. cuestensi* and *H. caimani*, respectively.

### Statistical analysis

Gamont measurements were recorded from *B. raniceps*, *T. typhonius*, *R. diptycha*, *L. labyrinthicus*, *L. macrosternum*, and *A. ameiva*, but were excluded from statistical analysis due to insufficient morphotype representation per sample. In a homogeneous sample, no more than 5% of observations should fall outside the expected distribution; this criterion was met only in the *C. yacare* group. In contrast, outliers were detected in *B. constrictor* (n = 1), *E. notaeus* (n = 8), and *E. crassus* (n = 1). Full statistical outputs are provided in Supplementary Table 2.

### Molecular and Phylogenetic Analysis

A total of 142 out of 172 DNA samples (82.5%) successfully amplified the endogenous 16S rRNA gene, including 119 blood samples and 23 spleen samples. The spleen DNA samples consisted of six *B. raniceps*, ten *L. macrosternum*, two *T. typhonius*, two *L. labyrinthicus*, two *O. taurinus*, and one *R. diptycha*.

Overall, 33 of 142 samples (23.2%; 95% CI: 16.3–30.2%) were PCR-positive for *Hepatozoon* spp., including 27 blood and 6 spleen samples (Table 1). Six samples yielded high-quality 18S rRNA sequences: *R. diptycha* (GenBank^®^: PQ204671), *A. ameiva* (PQ204673), *B. constrictor* (PQ204674), *E. crassus* (PQ204676), *E. notaeus* (PQ204677), and *C. yacare* (PQ204672). Remaining PCR-positive samples could not be sequenced, likely due to DNA degradation during phenol-chloroform extraction. Only chromatograms with clear peaks and full-length coverage were retained for phylogenetic inference. The relationships among specimens, smear results, and PCR data are summarized in Table 1, and BLASTn results are presented in Table 2.

**Table 2:** Percentage of BLASTn-associated identity of sequences of the 18S rRNA gene of *Hepatozoon* spp. detected in amphibians and reptiles from the state of Mato Grosso, Brazil.

**Agents**	**Animal (ID)**	**Accession Number**	**Query Cover**	**E-Value**	**Identity**	**GenBank^®^ (Accession number)**
*Hepatazoon* sp.	*Rhinella diptycha* (ARV 69)	PQ204675	100%	0.0	99.92%	Similarity with *Hepatozoon musa* (KX880079)
*Hepatazoon* sp.	*Ameiva ameiva* (ARV 39)	PQ204673	100%	0.0	98.90%	Similarity with *Hepatozoon cuestensis* (ON2374591)
*Hepatazoon* sp.	*Boa constrictor (*ARV 44)	PQ204674	98%	0.0	99.7%	Similarity with *Hepatozoon cevapii* (ON237359)
*Hepatazoon* sp.	*Epicrates crassus* (ARV 71)	PQ204676	100%	0.0	99.93%	Similarity with *H. musa* (KX880079)
*Hepatazoon* sp.	*Eunectes notaeus* (NT 29)	PQ204677	100%	0.0	99.2%	Similarity with *H. musa* (KX880079)
*Hepatazoon* sp.	*Caiman yacare* (J17)	PQ204672	100%	0.0	99.11%	Similarity with *Hepatozoon* sp. (PP708609)

**Figure 3:**
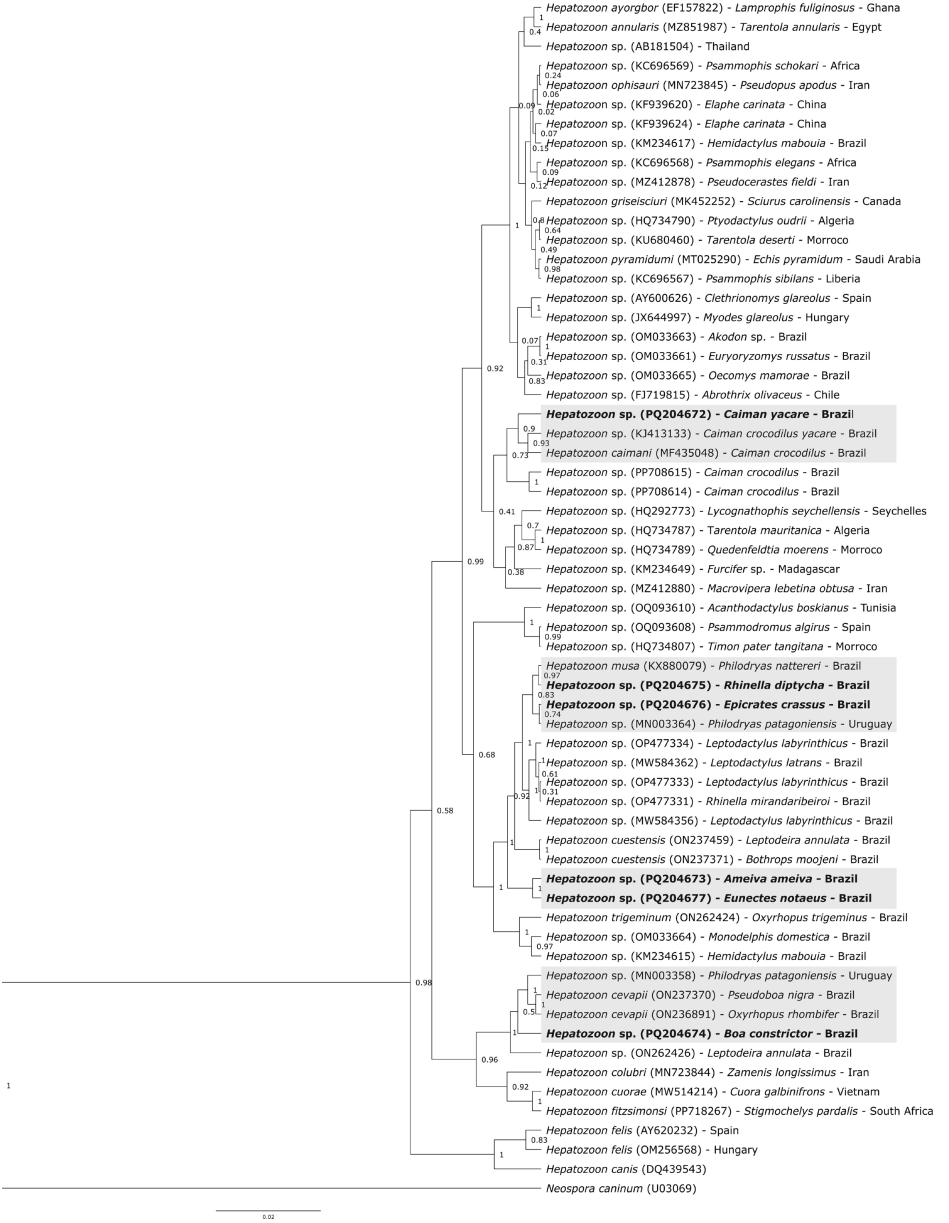
Phylogenetic analysis of partial 18S rRNA gene sequences from *Hepatozoon* spp. based on a 1,259 bp alignment using Bayesian inference, with GTR + G as an evolutionary model. The tree was rooted with *Neospora caninum* (UO0369). The sequences generated in the present study are written in **bold**, and the clades are highlighted in gray. Bayesian phylogenetic tree based on a 1,259 bp alignment of partial 18S rRNA gene sequences from *Hepatozoon* spp., using the GTR + G model of nucleotide substitution. The tree is rooted with *Neospora caninum* (UO0369). Sequences generated in this study are indicated in bold; major clades are shaded in gray.

Phylogenetic analysis revealed four distinct clades (Figure 3). The *C. yacare* sequence (PP708600) clustered with other *Hepatozoon* sequences from Brazilian crocodilians, forming a well-supported crocodilian-specific lineage. The second clade grouped *R. diptycha* (PQ204675) with *P. nattereri* (KX880079), and *E. crassus* (PQ204676) with *Philodryas patagoniensis* (MN003364) in two subclades (posterior probability < 0.74). The third clade included sequences from *A. ameiva* (PQ204673) and *E. notaeus* (PQ204677), clustering with *Hepatozoon* spp. from other Brazilian reptiles and amphibians (posterior probability = 1.0). The fourth clade contained *B. constrictor* (PQ204674), grouped with *Hepatozoon* spp. from Brazilian and Uruguayan snakes (posterior probability = 1.0).

## Discussion

In this study, representatives from three amphibian families and seven families within the order *Squamata* were screened for haemogregarine parasites using both Giemsa-stained blood smears and molecular diagnostics. The results from microscopic analysis (positive blood smears) and molecular testing (PCR-positive cases) were consistent with existing literature on test sensitivity (Mathew et al., 2000; Moço et al., 2002; Harris et al., 2011). In some cases, parasitemia was not detectable by microscopy but was confirmed through PCR, highlighting the enhanced sensitivity of molecular tools in identifying subpatent infections.

We additionally report new host records of *Hepatozoon* infections and describe six novel haplotypes within the genus. While *Hepatozoon* species are commonly reported in reptiles—especially snakes—anurans are also known to harbor diverse hemoparasites. This study documents, for the first time, *Hepatozoon* infection in the anurans *B. raniceps* and *T. typhonius*, as well as in the snake *E. notaeus*.

Trophic transmission via predator-prey interactions represents a key pathway for sustaining multi-host *Hepatozoon* lineages and driving coevolutionary dynamics. Host ecology plays a critical role in shaping parasite exposure and transmission potential (Tomé et al., 2012). For instance, species with arboreal, terrestrial, or aquatic lifestyles encounter vectors and infected prey differently within the food web. While prior studies have shown higher hemogregarine prevalence in semi-aquatic amphibians (Netherlands et al., 2015), our findings revealed elevated infection rates in arboreal (*B. raniceps*, *T. typhonius*) and terrestrial (*R. diptycha*, *L. labyrinthicus*, *L. macrosternum*) species, although parasitemia remained low in these individuals.

A similar trend was observed in reptiles, with positive cases detected in terrestrial species (*A. ameiva*, *B. constrictor*, *E. crassus*) and semi-aquatic species (*E. notaeus*, *C. yacare*), all displaying relatively low parasitemia. Species that co-inhabit ecotones such as riverbanks or forest edges may serve as ecological bridges for parasite dispersal across niches (Borremans et al., 2019). These overlaps may support generalist *Hepatozoon* lineages with low vertebrate host specificity, possibly leading to “dead-end” infections in terminal predators and contributing to dilution effects (Thomas et al., 2024). This hypothesis is reinforced by the clustering of some haplotypes with sequences from phylogenetically distant hosts, suggesting that ecological factors rather than host phylogeny shape infection patterns.

The identification of developmental stages in both prey and predator hosts indicates that *Hepatozoon* spp. possess a complex heteroxenous life cycle (Smith, 1996). Such cycles typically involve a hematophagous invertebrate definitive host, a vertebrate intermediate host, and in some cases, a paratenic host that facilitates trophic transfer. These complexities complicate the definition of a “typical” parasite morphology, which may vary depending on host species. Generally, *Hepatozoon* species infecting hosts within the same biome or habitat type display higher occurrence rates (Brian & Aldridge, 2024), but cross-biome comparisons are often limited by challenges in obtaining representative samples (Clemente et al., 2023). The multiple prey-predator infections observed in this, and other studies (Harris et al., 2011) highlight the need for integrative approaches that combine host ecology, dietary reconstruction (e.g., DNA-based diet analysis), and experimental infections to better understand host specificity, breadth, and coevolution in *Hepatozoon* transmission systems.

Observer bias can significantly influence the interpretation of microscopic hemoparasite observations, affecting both the clarity and reliability of diagnostic conclusions (Thompson, 2009). Therefore, incorporating tools that mitigate this bias—such as image measurement software and morphometric statistics—is essential. In this study, ImageJ software and Mahalanobis distance analysis were used to quantify and differentiate morphotypes. Comparing findings with established literature and applying validated methodologies further strengthened the diagnostic consistency, allowing results to be interpreted in a comparative context.

Although several morphotypes were identified, the congruence between morphological characteristics and molecular groupings was inconsistent in some cases. Consequently, no formal taxonomic proposals were made. The newly described forms are herein considered preliminary and foundational references for future research in the respective host species.

The genus *Hepatozoon* is well known for its pronounced morphological plasticity and broad host range (Ball, 1970; Smith, 1996; Tomé et al., 2012; Netherlands et al., 2015; Paperna & Lainson, 2003), enabling it to adapt to different host cell types and parasitic niches. The morphometric results from this study revealed a broader spectrum of morphological variation than previously documented for *H. musa* (O'Dwyer et al., 2013), *H. cevapii* (Úngari et al., 2022), and *H. caimani* (Lainson et al., 2003; Clemente et al., 2023). However, as observed herein, despite the separation of groups into morphotypes, the presence of a heterogeneous group, evidenced by outlier values over 5%, further suggests that visual identification alone may not be a reliable method for distinguishing parasites exhibiting significant morphological plasticity. The differences observed may be associated with specific adaptations of the parasites to their respective hosts.

Additionally, the identification of six unique 18S rRNA haplotypes within a 23.2% PCR positivity rate points to considerable intra-regional or intra-host diversity. This supports the hypothesis that morphologically similar *Hepatozoon* forms may represent cryptic, genetically distinct lineages (Úngari et al., 2024). Although quantitative morphometric data supported the morphotype classifications, overlaps between morphology and molecular phylogeny suggest that morphology alone does not offer robust species-level resolution. This finding underscores the importance of an integrative taxonomic approach that combines molecular, morphological, and ecological data for effective species delimitation in *Hepatozoon*.

The morphotypes found in *R. diptycha* do not resemble those reported by Carvalho et al. (2024) in species of the *Rhinella* genus, furthermore, that study lacked molecular analysis for comparative validation. However, it resembles the form of *H. longinucleus* sp. nov. (Úngari et al., 2021), although nuclear and cytoplasmic characteristics are different from those reported in the literature. The morphometric comparison between *H. longinucleus* sp. nov., *Hepatozoon* sp. (Carvalho et al., 2024), and from the present work reveals significant variations in the analyzed parameters. Although none of the works present all the comparable morphotypes, the comparison between the mature gamonts present in the three works, *H. longinucleus* sp. nov.*,* shows greater similarity with the minimum values of length and area observed and is therefore the closest morphometrically. The differences found reinforce the idea of the parasite's ability to adapt to the host's cells and may present subtle differences in its morphometry and highlight the importance of integrating morphological and molecular data for adequate taxonomic delimitation.

Among reptiles, despite a wide range of described species (Picelli et al., 2020), many accounts remain taxonomically incomplete, limiting comparative analyses. The morphotypes identified in *A. ameiva* in this study exhibit morphological resemblance to *H. ameivae* (Picelli et al., 2020), the most frequently reported *Hepatozoon* species in this host. However, they differ from *H. cuestensi* (O'Dwyer et al., 2013), which emerged as a phylogenetically close lineage in this dataset. These comparisons suggest that the observed morphotype may align more closely with *H. ameivae*, but further validation, including molecular confirmation, remains necessary.

Herein, the *Hepatozoon* morphotype identified in *A. ameiva*, along with its phylogenetic placement, does not correspond with previously reported morphological descriptions for lizards from Central Amazonia (Picelli et al., 2020). For lizards, molecular data are available for all described *Hepatozoon* species, including *H. tupinambis* in *Tupinambis teguixim* (Laveran & Salimbeni, 1909), *H. missoni* in *T. teguixim* (Carini, 1909a), *H. ameivae* in *Ameiva* sp. (Carini & Rudolph, 1912), *H. cnemidophori* in *Cnemidophorus* sp. (Carini, 1941a, b), and *H. sinimbui* in *Iguana iguana* (Picelli et al., 2020).

The *Hepatozoon* morphotypes observed in the blood smear of *B. constrictor* were morphologically similar to *H. cevapii* (Úngari et al., 2022), a relationship confirmed by phylogenetic analysis. Morphometric comparisons between the gamonts of *H. cevapii* revealed that the specimen has a length compatible with the range observed in the present study but exhibits a substantially narrower width. The parasite’s area is also smaller, although still within the observed range. These differences suggest that, despite similar length, the narrower width and smaller area indicate that the morphotypes may represent a haplotype distinct from that originally described.

In *E. notaeus*, the observed *Hepatozoon* morphotypes showed morphological similarities to *H. cuestensi* (O'Dwyer et al., 2013), a relationship also supported by phylogenetic analysis. Comparisons between the gamont morphotypes of *H. cuestensi* and those described in this study reveal strong morphometric compatibility. All values from the former fall within the ranges observed in the latter, particularly for length and area. Although the width of *H. cuestensi* is slightly lower than the group average, the variation is minor and does not undermine compatibility. These findings suggest that the gamonts examined may belong to the same morphological group, with low intraspecific variation. This study also provides a morphometric description of a parasitic form not previously identified in this species—the macrogamont (M22)—and the first record of *Hepatozoon* infection in this host.

In contrast, the *Hepatozoon* morphotypes identified in *E. crassus* are closely aligned with previously reported morphological descriptions of *H. musa* (Borges-Nojosa et al., 2017), initially recorded in this host by Úngari et al. (2018). The morphometric values of gamonts described by Borges-Nojosa et al. (2017) fall within the ranges reported in this study. Although the width is closer to the lower limit and the length approximates the group mean, the area closely corresponds to the estimated mean. These similarities suggest that the parasites described in this host may represent a subgroup of those identified by Borges-Nojosa et al. (2017).

Crocodilians have been extensively studied for *Hepatozoon* infections; however, in Brazil, *H. caimani* remains the only valid *Hepatozoon* species described in native caimans (Úngari et al., 2024). Recent findings, though, raise questions about the diversity of haplotypes and morphotypes within this group (Clemente et al., 2023). The morphotype identified herein displays distinct morphological features compared to those previously reported for *H. caimani* in Brazil (Carini, 1909b). However, in terms of measurements, it aligns with values documented by Bouer et al. (2017) and Clemente et al. (2023), although its phylogeny clusters with other *Hepatozoon* species described in Brazilian crocodilians. Our data supports previous research demonstrating both morphological and haplotypic diversity in this group (Clemente et al., 2023). Furthermore, no outliers were detected among the crocodilian samples in this study, reinforcing the observed homogeneity.

For species where molecular data could not be obtained, only morphological comparisons were possible. In *B. raniceps*, the morphotypes observed resembled those reported in *L. labyrinthicus*, which were classified as *Hepatozoon longinucleus* sp. nov. (Úngari et al., 2021). While both exhibited similarly rounded ends (with one end slightly tapered and curved) and thin parasitophorous capsules, morphometric comparison revealed marked differences. The notable morphological differences exhibited by the current morphotype indicate a distinct morphometric profile, suggesting taxonomic distinction. This reinforces the importance of combining morphometry with other diagnostic parameters in the classification of hemogregarines, particularly where only gamont stages are available and no prior host infection records exist.

The morphotype identified in *T. typhonius* does not match any known records in Brazilian amphibians (Carini, 1908; Costa et al., 1973; Úngari et al., 2021; Carvalho et al., 2024). Its mature gamont presented characteristics closest to *H. leptodactyli* described by Carini (1908), with mean dimensions comparable to those of morphotype M6, however, the author does not describe which parasitic form is described. Additionally, the absence of genetic information in this case complicates comparison through alternative methods and hinders the detailed description of the parasite. Still, this is the first description of infection by the genus *Hepatozoon* in this host species.

Moreover, the *Hepatozoon* sp. identified in *L. labyrinthicus* exhibited morphological resemblance to *Hepatozoon formosus* (Úngari et al., 2021). However, the absence of additional developmental stages in the present study limits deeper taxonomic comparisons between the groups. Morphometric analyses of immature gamonts from *H. formosus* and from morphotypes M11 and M12 in this study revealed pronounced differences. In contrast, the immature gamonts observed herein were significantly larger across all morphometric parameters, with no overlap in value ranges. These distinctions suggest that the two morphotype groups are taxonomically distinct, underscoring the need for integrative approaches in species delimitation. Nonetheless, due to the absence of other parasitic stages, comparisons in this study were confined to immature gamonts.

In the case of the *L. macrosternum* specimen, the observed morphology closely resembled that of *Hepatozoon* sp. described by Leal et al. (2015). A comparison of the morphometric parameters of mature gamont morphotype M15 with previously described anuran species indicated the greatest similarity with *H. leptodactyli*, particularly as described by Carini (1908). This suggests that M15 may represent a morphological variant of this species.

A key limitation of this study was the low sequencing success among PCR-positive samples, with only 6 out of 33 amplifications yielding usable sequences. This may be attributed to DNA degradation, potentially linked to phenol-chloroform extraction (Kramvis et al., 1996). Consequently, phylogenetic resolution was limited (Hufnagl et al., 2013), constraining our capacity to detect parasite diversity. To overcome these issues, future studies may adopt commercial DNA extraction kits, which offer improved recovery of high-quality DNA, despite requiring protocol adaptations due to nucleated cells in reptilian blood. Additionally, incorporating complementary molecular markers such as the nuclear ribosomal ITS-1 or mitochondrial *cox1* genes—which are commonly used to differentiate closely related taxa and enhance phylogenetic inference—could prove valuable, although such markers remain limited in hemoprotozoans. These methodological enhancements are expected to strengthen the depth and accuracy of future molecular investigations.

In this context, 18S rDNA gene sequence comparisons remain a valuable tool for species identification (Sambrook & Russell, 2001) due to their wide availability in public databases and ability to support broad-scale phylogenetic analyses. However, caution is warranted. The current *Hepatozoidae* database is inconsistent, which can lead to poorly resolved or weakly supported phylogenies (Young & Gillung, 2020). Two primary databases exist for these sequences (Guillou et al., 2013; Quast et al., 2013): GenBank^®^, which contains numerous misidentified entries (Balvočiūtė & Huson, 2017), and the PR2 database, which has been partially corrected through expert curation (Campo et al., 2018). These issues likely stem from early submissions made when reference frameworks were incomplete, and the phylogenetic classification of Apicomplexa remains challenging.

Phylogenetic analyses of partial 18S rDNA sequences from *Hepatozoon* sp. infecting Brazilian amphibians and reptiles suggest that some lineages may lack strict host specificity and instead circulate across multiple host species. This supports the hypothesis of trophic transmission, where infections arise from predator-prey interactions within complex food webs (Harris et al., 2011). As a result, associations with intermediate hosts are becoming more apparent in recent studies of *Hepatozoon* transmission. Notably, species infecting predators that feed primarily on anurans tend to form phylogenetically distinct lineages from those infecting predators with predominantly saurian diets (Tomé et al., 2012).

In contrast, despite the high reptile biodiversity in Brazil, only five snake species have available *Hepatozoon* sequences (Úngari et al., 2022): *H. cevapii* in *Crotalus durissus* (O'Dwyer et al., 2013) and *Thamnodynastes lanei* (Paula et al., 2021); *H. cuestensis* and *H. massardii* in *C. durissus* (O'Dwyer et al., 2013); *H. musa* in *P. nattereri*, *C. durissus*, and *E. crassus* (Moço et al., 2002); and *H. quagliattus* in *Dipsas mikanii* (Úngari et al., 2018).

Furthermore, Brazilian studies often report strong phylogenetic affinities between local *Hepatozoon* species and African strains, particularly in amphibians (Úngari et al., 2021)—a pattern not observed in this study. This discrepancy underscores the need for broader and higher-quality molecular datasets, especially those comparable to existing 18S rDNA data for haemogregarines. Expanding such datasets is essential for accurately characterizing host diversity within the herpetofauna and for clarifying the biogeographical patterns of *Hepatozoon* sp.

## Conclusions

In conclusion, infections by *Hepatozoon* sp. were documented for the first time in three species in Brazil: the snake *E. notaeus* and the anurans *B. raniceps* and *T. typhonius*. These findings expand the known host range of the *Hepatozoon* genus in Brazil. Finally, this study contributes to a broader understanding of parasitic and ecological dynamics in the region and highlights the importance of combining molecular and morphological data to distinguish *Hepatozoon* variants. The results also suggest that genetic diversity within the genus may be greater than currently recognized.

## Supplementary Material

Supplementary material accompanies this paper.

Supplementary Table 1. Measurements and characteristics of the *Hepatozoon* morphotypes found in the anurans *Boana raniceps*, *Trachycephalus typhonius*, *Rhinella diptycha*, *Leptodactylus labyrinthicus,* and *Leptodactylus macrosternum*; in the lizard *Ameiva ameiva*; in the snakes *Boa constrictor, Eunectes notaeus,* and *Epicrates crassus*; and the crocodilian *Caiman yacare*, Mato Grosso State, Brazil.

Supplementary Table 2. Measurements of the *Hepatozoon* gamonts (area, perimeter, and maximum and minimum Feret diameters).

This material is available as part of the online article from https://doi.org/10.1590/S1984-29612025076
