# Supplementary material for: Morphological and molecular detection of Hepatozoon species in amphibians and reptiles from Mato Grosso, Midwest Brazil
Source: Rev Bras Parasitol Vet. 2026 Feb 2;34(4):e012125. doi: 10.1590/S1984-29612025076 (PMC12974785; doi:10.1590/S1984-29612025076)
Supplement: Supplementary Table 1. [file rbpv-34-4-e012125-suppl1.pdf]

Suplementary Table 1. Measurements and characteristics of the *Hepatozoon* morphotypes found in the anurans *Boana raniceps*, *Trachycephalus typhonius*, *Rhinella diptycha*, *Leptodactylus labyrinthicus,* and *Leptodactylus macrosternum*; in the lizard *Ameiva ameiva*; in the snakes *Boa constrictor, Eunectes notaeus,* and *Epicrates crassus*; and the crocodilian *Caiman yacare*, Mato Grosso State, Brazil.

Taxonomic group	M[1]	Area[2]	P[3]	Mx[4]	Mn[5]	Morphology
**Anura**						
**Hylidae**						
	**M1 - Immature gamont**					Slender, with one domed and one tapered end. Observation of basophilic granules and vacuoles in the cytoplasm. Dense and centralized nucleus. The capsule is not evident. The parasite is located close to the nucleus.
	Parasite	21.52-23.41 ±1.33	24.8-31.14 ±4.48	10.55-11.40 ±0.60	3.03-4.20 ±0.82	
	Nuclei	7.99-11.94 ±2.79	11.33-16.24 ±3.47	4.00-6.67 ±1.89	2.46-2.62 ±0.11	
	**M2 - Immature gamont**					Small and robust, with bulging ends. Observation of basophilic granules and vacuoles in the cytoplasm. Dense and centralized nucleus. The capsule is not evident. The parasite is located next to the nucleus.
Boana raniceps	Parasite	52.22	31.25	11.61	5.41	
	Nuclei	9.71	11.62	4.12	3.16	
	**M3 - Mature gamont**					Elongated and thin, with a dense and centralized nucleus. With slightly basophilic granules dispersed in the cytoplasm. The capsule is evident. The parasite is closely related to the nucleus (embraced)
	Parasite	42.59-66.05 ±5.97	39.42-54.97 ±4.08	15.42-19.05 ±0.84	4.71-6.96 ±0.54	
	Nuclei	7.50-16.13 ±2.23	11.28-19.89 ±1.74	3.58-7.65 ±0.78	2.04-3.80 ±0.49	
	**M4 - Mature gamont**					

Taxonomic group	M[1]	Area[2]	P[3]	Mx[4]	Mn[5]	Morphology
	Parasite	43.25-69.85 ±6.39	37.91-50.81 ±2.64	15.55-18.38 ±0.69	4.66-7.03 ±0.59	Elongated and thin, with one end domed and the other with a curvature (hook shape). Observation of slightly basophilic granules dispersed in the cytoplasm. The capsule is evident. The parasite is closely related to the nucleus (embraced).
	Nuclei	3.47-13.97 ±2.41	9.98-16.59 ±1.67	3.93-6.72 ±0.72	1.28-3.56 ±0.49	
	**M5 - Immature gamont**					Small and slender with tapered ends. Observation of granules and cytoplasmic vacuoles. Nucleus elongated and centralized. The capsule is not evident. The parasite is located next to the nucleus.
	Parasite	5.72-6.53 ±0.40	10.55-12.24 ±0.92	4.04-5.31 ±0.64	1.59-2.08 ±0.25	
	Nuclei	1.73-3.21 ±0.77	5.39-9.38 ±2.24	2.22-4.09 ±0.93	1.09-1.45 ±0.19	
	**M6 - Mature gamont**					Robust and long, with domed ends. Observation of basophilic granules and vacuoles in the cytoplasm. Centralized nucleus. The capsule is evident. The parasite is closely related to the nucleus (embraced).
	Parasite	42.26-78.08 ±18.09	31.36-45.33 ±7.16	12.29-16.93 ±2.46	5.32-6.44 ±0.55	
Trachcephalus typhonius	Nuclei	10.00-16.21 ±3.15	15.73-16.56 ±0.52	6.10-6.29 ±0.11	3.15-3.44 ±0.70	
	**M7 – Macrogamont**					Large and robust, with bulging ends, occupying practically the entire cytoplasm of the host cell. Observation of basophilic granules scattered throughout the cytoplasm. Large and centralized nucleus. Scattered chromatin. The capsule is evident. The parasite is closely related to the nucleus (embraced).
	Parasite	35.26-47.78 ±4.21	34.15-37.17 ±1.15	12.34-13.92 ±0.60	5.20-5.88 ±0.22	
	Nuclei	9.49-24.93 ±6.04	14.26-22.75 ±3.44	6.12-9.16 ±1.36	1.85-3.64 ±0.65	
**Bufonidae**						

Taxonomic group	M[1]	Area[2]	P[3]	Mx[4]	Mn[5]	Morphology
Rhinella diptycha	**M8 - Immature gamont**					Small, with slightly tapered ends. Observation of cytoplasmic vacuoles. Small nucleus and located at one of the ends. The capsule is not evident. The parasite is located next to the nucleus.
	Parasite	40.12-183.46 ±65.30	30.73-54.32 ±11.33	12.13-18.10 ±3.67	5.01-11.43 ±3.19	
	Nuclei	10.06-39.13 ±7.50	12.04-27.42 ±2.32	4.55-10.44 ±1.58	3.16-6.00 ±0.97	
	**M9 - Mature gamont**					Long, with bulging ends (hook shape). Observation of granules and vacuoles in the cytoplasm. Dense, quadrangular nucleus, central. The capsule is evident. The parasite is located far from the nucleus.
	Parasite	25.28-275.54 ±93.33	27.63-70.00 ±14.82	10.77-26.06 ±5.57	3.00-13.86 ±3.99	
	Nuclei	3.17-32.19 ±12.16	7.55-30.30 ±7.70	2.65-11.33 ±3.06	2.61-4.11 ±0.93	
	**M10 – Macrogamont**					Large, robust, with bulging ends. Observation of a few cytoplasmic vacuoles. Large, central nucleus. The capsule is evident. The parasite is closely related to the nucleus (embraced).
	Parasite	124.32-302.22 ±99.32	46.83-73.43 ±14.86	17.14-23.29 ±3.21	9.96-18.28 ±4.79	
	Nuclei	25.83-40.23 ±7.30	21.71-30.91 ±5.06	7.36-11.36 ±2.12	4.90-5.98 ±0.57	
**Leptodactylidae**						
Leptodactylus labyrinthicus	**M11 - Immature gamont**					Robust, with one end bulging and the other tapered. The nucleus occupies more than 50% of the cytoplasm. Observation of cytoplasmic vacuoles. The capsule is not evident. The parasite is located next to the nucleus.
	Parasite	121.25-130.75 ±6.35	58.11-62.87 ±3.36	24.58-26.49 ±1.34	6.92-7.61 ±0.49	
	Nuclei	67.70-73.96 ±4.42	37.62-41.92 ±0.83	15.09-16.82 ±3.04	5.60-5.90 ±0.20	
	**M12 - Immature gamont**					

Taxonomic group	M[1]	Area[2]	P[3]	Mx[4]	Mn[5]	Morphology
	Parasite	86.30-88.91 ±1.84	40.39-40.88 ±0.34	16.27-16.51 ±0.16	6.76-6.95 ±0.13	Robust and small. Observation of cytoplasmic vacuoles at the ends. The nucleus occupies more than 50% of the cytoplasm. The capsule is not evident. The parasite is located next to the nucleus.
	Nuclei	58.58-66.47 ±5.58	28.64-31.76 ±2.20	10.24-11.90 ±1.17	7.07-7.11 ±0.03	
Leptodactylus macrosternum	**M13 - Immature gamont**					
	Parasite	3.72-9.90 ±1.65	9.42-16.43 ±1.80	4.02-7.06 ±0.73	1.21-2.19 ±0.25	Robust, with both ends bulging; however, one side is wider than the other. It is possible to observe vacuoles spread throughout the cytoplasm. Dense core and occupies one of the ends. The capsule is evident. The parasite is located close to the nucleus of the red blood cell.
	Nuclei	0.80-4.49 ±1.22	3.86-10.81 ±2.35	4.40-4.40 ±1.06	0.90-1.80 ±0.26	
	**M14 - Immature gamont**					
	Parasite	9.13-16.03 ±1.90	13.16-17.37 ±1.30	5.32-7.09 ±0.50	2.34-3.09 ±0.23	Large, robust, with bulging ends. Observation of granules and vacuoles in the cytoplasm. The nucleus is large, slightly dislocated to one end. Loose chromatin. The capsule is evident. The parasite is located next to the nucleus.
	Nuclei	3.71-5.65 ±0.75	6.93-10.05 ±1.01	2.86-3.86 ±0.32	2.06-2.52 ±0.17	
	**M15 - Mature gamont**					
	Parasite	6.28-11.13 ±1.52	13.13-16.29 ±1.26	5.11-6.56 ±0.54	1.87-2.60 ±0.24	Elongated, with bulging ends. The nucleus is small and located at one of the ends. The capsule is evident. The parasite is located next to the nucleus.
	Nuclei	1.76-5.57 ±1.34	5.48-11.43 ±1.83	1.74-3.84 ±0.76	1.26-2.06 ±0.24	
**Squamata**						

Taxonomic group	M[1]	Area[2]	P[3]	Mx[4]	Mn[5]	Morphology
**Teiidae**						
Ameiva ameiva	**M16 - Immature gamont**					Robust and small with domed ends. Observation of vacuoles in the cytoplasm. Small, dense nucleus located at one of the ends. The capsule is not evident. The parasite is located next to the nucleus.
	Parasite	21.08-21.88 ±1.15	20.60-24.36 ±1.93	8.07-8.98 ±0.44	3.41-3.69 ±0.13	
	Nuclei	5.3-7.15 ±0.90	9.08-12.16 ±1.61	3.53-8.89 ±0.39	2.15-2.70 ±0.27	
	**M17 - Mature gamont**					Robust and small with domed ends. Observation of basophilic granules distributed in one of the ends. Small, dense nucleus located at the ends. The capsule is evident. The parasite is located next to the nucleus.
	Parasite	29.867	25.46	9.72	4.29	
	Nuclei	7.80	12.30	4.58	2.36	
**Boidae**						
Boa constrictor	**M18 - Immature gamont**					The shape is close to piriform. Observation of vacuoles in the cytoplasm. The nucleus is dispersed and central. The capsule is evident. The parasite is located next to the nucleus.
	Parasite	12.71-24.38 ±3.71	17.66-22.69 ±1.57	7.25-8.42 ±0.40	2.13-3.57 ±0.46	
	Nuclei	1.38-4.01 ±1.12	5.86-14.01 ±2.33	1.96-5.22 ±1.03	0.93-2.25 ±0.37	
	**M19 - Mature gamont**					Robust, with both domed ends. The nucleus is dense, quadrangular, and central. The capsule is evident. The parasite is closely related to the nucleus (embraced).
	Parasite	31.33-224.3 ±67.21	28.52-57.46 ±8.82	10.78-23.77 ±3.77	3.97-13.05 ±2.96	
	Nuclei	6.72-23.94 ±5.47	10.20-21.63 ±2.78	3.86-9.01 ±1.48	2.00-4.69 ±0.68	
	**M20 – Macrogamont**					Robust, elliptical, occupying practically the entire cytoplasm of the host cell. Observation
	Parasite	96.91-406.16 ±131.45	43.20-86.89 ±18.11	17.08-29.27 ±5.20	7.05-19.41 ±5.12	

Taxonomic group	M[1]	Area[2]	P[3]	Mx[4]	Mn[5]	Morphology
	Nuclei	21.52-47.40 ±11.59	18.93-32.37 ±6.10	6.31-13.33 ±3.00	4.70-5.55 ±0.31	of basophilic granules distributed throughout the cytoplasm. The nucleus is rounded and centralized. The capsule is evident. The parasite pushes the nucleus.
Eunectes notaeus	**M21 - Mature gamont**					Elongated, with both ends bulging with a slight curvature. The nucleus is dense and dislocated in one of the poles. The capsule is evident. The parasite is closely related to the nucleus (embraced).
	Parasite	18.46-96.98 ±17.20	25.91-49.25 ±21,65	10.46-17.71 ±1.30	3.20-8.09 ±1.13	
	Nuclei	4.60-20.92 ±14.68	10.05-27.45 ±2.67	3.66-12.63 ±2.91	1.17-4.67 ±0.87	
	**M22 – Macrogamont**					Robust, occupying a large part of the host cell cytoplasm. Observation of cytoplasmic vacuoles distributed throughout the entire size of the parasite (lace-like appearance). The nucleus is dense, elliptical, and close to one of the ends. The capsule is evident. The parasite pushes the nucleus.
	Parasite	16.93-155.62 ±18.19	15.34-50.58 ±4.36	5.41-21.79 ±1.32	3.11-9.73 ±1.15	
	Nuclei	16.90-287.04 ±3.58	14.94-60.74 ±2.69	4.75-60.74 ±29.19	2.97-16.93 ±0.88	
Epicrates crassus	**M23 - Immature gamont**					Slender, with slightly curved ends. Observation of cytoplasmic vacuoles and granules. The nucleus is long, misshapen, and approaches one of the ends. The capsule is evident. The parasite is located next to the nucleus.
	Parasite	10.83-18.64±3,73	50.1-66.91 ±24.53	19.28-23.23 ±9.28	8.63-10.91 ±2.21	
	Nuclei	1.92-7.02 ±1.87	20.65-39.41 ±28.05	7.38-14.05 ±11.73	3.26-6.80 ±5.34	
	**M24 - Mature gamont**					Slender, with slightly curved ends (hook shape). The nucleus is rounded and central. The capsule is evident. The parasite is located next to the nucleus.
	Parasite	25.85-203.20 ±26.9	19.72-88.92 ±4.54	7.27-30.47 ±1.37	3.58-11.94 ±9.8	
	Nuclei	15.11-539.84 ±14.17	16.82-90.24 ±4.36	6.44-38.00 ±2.03	2.83-20.12 ±1.16	

Taxonomic group	M[1]	Area[2]	P[3]	Mx[4]	Mn[5]	Morphology
**Crocodylia**						
**Alligatoridae**						
	**M25 - Immature gamont**					Small, with one domed and the other tapered end. The nucleus is large, triangular, and located closer to one of the ends. The capsule is not evident. The parasite is located next to the nucleus.
	Parasite	45.79-47.92 ±1.51	28.91-31.54 ±1.82	12.26-12.62 ±0.25	4.81-4.86 ±0.03	
	Nuclei	18.72-19.33 ±0.43	19.2-18.27 ±0.65	6.69-6.76 ±0.05	4.16-4.51 ±0.25	
	**M26 - Mature gamont**					Elongated, with both bulging ends. Observation of granules and vacuoles in the cytoplasm. The nucleus is thin and elongated. The capsule is evident. The parasite is located next to the nucleus.
	Parasite	31.04-67.53 ±10.45	28.61-34.20 ±1.83	11.10-13.73 ±0.77	4.41-6.65 ±0.72	
Caiman yacare	Nuclei	3.57-19.77 ±4.86	10.68-20.73 ±3.17	4.84-7.97 ±1.15	0.98-4.43 ±1.13	
	**M27 – Macrogamont**					Large and robust, with both bulging ends, it occupies almost the entire cytoplasm of the host cell. Observation of a few granules and vacuoles scattered in the cytoplasm. The nucleus is large, rectangular, and occupies almost the entire length of the parasite, except for the poles. The capsule is evident. The parasite pushes the nucleus.
	Parasite	83.83-119.32 ± 25.1	38.54-43.32 ±3.38	15.96-18.34 ±1.69	6.92-8.28 ±0.96	
	Nuclei	14.59-56.38 ±29.55	21.70-26.63 ±3.48	8.48-9.48 ±0.71	2.51-8.48 ±4.22	

[1]Morphotype; [2]Area ±Stand Deviation.; [3]Perimeter ±Stand Deviation; [4]Maximum Feret ±Stand Deviation; [5]Minimum Feret ±Stand Deviation
