# Supplementary material for: Morphological and molecular detection of Hepatozoon species in amphibians and reptiles from Mato Grosso, Midwest Brazil
Source: Rev Bras Parasitol Vet. 2026 Feb 2;34(4):e012125. doi: 10.1590/S1984-29612025076 (PMC12974785; doi:10.1590/S1984-29612025076)
Supplement: Supplementary Table 2. [file rbpv-34-4-e012125-suppl2.pdf]

Supplementary Table 2. Measurements of the *Hepatozoon* gamonts (area, perimeter, and maximum and minimum Feret diameters).

Specie	Measurements	N[1]	Mean	Standard deviation	CV (%)[2]	Minimum	Maximum	iqr[3]
Boa constrictor								
	**Mature gamont**							
	Area	16	118.65	67.21	56.65	31.34	224.3	110.93
	Perimeter	16	44.87	8.83	19.68	28.53	57.46	12.93
	Feret	16	18.4	3.77	20.51	10.78	23.78	5.19
	Minimum Feret	16	7.98	2.97	37.15	3.98	13.05	5.22
	**Parasite's nuclei**							
	Area	16	17.39	5.48	31.52	6.72	23.94	8.95
	Perimeter	16	16.39	2.79	17.02	10.2	21.63	2.86
	Feret	16	6.28	1.48	23.63	3.86	09.01	1.93
	Minimum Feret	16	3.63	0.68	18.82	2	4.7	0.69
Eunectes notaeus								
	**Mature gamont**							
	Area	86	56.62	18.19	32.13	18.46	96.99	29.32
	Perimeter	86	37.59	4.36	11.60	25.91	49.26	5.67
	Feret	86	15.03	1.32	8.76	10.41	17.72	1.61
	Minimum Feret	86	5.56	1.15	20.59	3.21	8.10	1.89
	**Parasite's nuclei**							
	Area	86	10.74	3.58	33.34	4.60	20.90	5.80
	Perimeter	86	10.04	2.69	19.18	10.06	27.45	2.12
	Feret	86	5.20	29.19	22.89	3.66	12.63	01.06
	Minimum Feret	86	3.02	0.88	29.19	1.17	4.68	1.35
Epicrates crassus								

Specie	Measurements	N[1]	Mean	Standard deviation	CV (%)[2]	Minimum	Maximum	iqr[3]
	**Mature gamont**							
	Area	34	100.79	62.18	61.69	25.86	203.21	117.42
	Perimeter	34	49.45	24.53	49.60	19.72	88.93	47.57
	Feret	34	18.80	9.28	49.32	7.27	30.47	18.95
	Minimum Feret	34	7.74	2.21	2.21	3.58	11.95	3.04
	**Parasite's nuclei**							
	Area	34	192.07	187.35	97.54	15.01	539.84	348.28
	Perimeter	34	53.46	28.05	52.46	16.82	90.25	52.89
	Feret	34	20.71	11.73	56.64	6.44	38.00	22.65
	Minimum Feret	34	10.37	5.34	51.52	2.84	20.13	8.50
Caiman yacare								
	**Mature gamont**							
	Area	11	44.03	10.45	23.74	31.04	67.54	12.07
	Perimeter	11	30.43	1.83	6.00	28.62	34.20	2.84
	Feret	11	12.62	0.77	6.09	11.11	13.74	0.90
	Minimum Feret	11	4.75	0.72	15.17	3.99	6.66	0.52
	**Parasite's nuclei**							
	Area	11	12.12	4.86	40.06	3.57	19.78	4.91
	Perimeter	11	14.94	3.17	21.25	10.68	20.74	3.93
	Feret	11	5.85	1.15	19.60	4.84	7.97	1.35
	Minimum Feret	11	2.98	1.13	37.80	0.98	4.43	1.53

[1]Number of gamonts measured; [2]Coefficient of variation; [3]Quartile range
